# Book Review: Efron, N. *Contact Lens Practice*; Elsevier Health Sciences: Amsterdam, The Netherlands, 2024; ISBN: 978-0-7020-8427-0

**DOI:** 10.3390/vision7040066

**Published:** 2023-10-11

**Authors:** Nir Erdinest

**Affiliations:** Department of Ophthalmology, Hadassah-Hebrew University Medical Center, Faculty of Medicine, Hebrew University of Jerusalem, Jerusalem 91120, Israel; nir.erdinest@mail.huji.ac.il; Tel.: +972-2-677-111

The book *Contact Lens Practice* (ISBN 978-0-7020-8427-0), fourth edition [1], was published in 2023 by Elsevier Health Science (Amsterdam, the Netherlands). The first, second, and third editions were copyrighted in 2002, 2010, and 2018, respectively. This comprehensive resource includes the contributions of 25 authors, for five of whom, this is their chapter debut, and was edited by Nathan Efron, a renowned expert in the field.

The book is organized into six sections, each comprising a varying number of chapters, culminating in forty chapters. This edition includes twelve practical, implementable appendices covering contact lens prescription writing, conversion tables, fitting records, patient instruction sheets, complication management charts, and infection management, all based on the high number of data in this field that are constantly accumulating, as evidenced by the volume of citations and references.

This edition has been thoroughly updated and revised; five chapters from previous editions have undergone complete reconstruction, and the chapter ‘Rigid Scleral and Corneoscleral Lens Design and Fitting’ is an entirely fresh addition. In contrast, chapters like ‘Planned Replacement Rigid Lenses’, ‘Scleral Lenses’, and ‘Digital Imaging’ have been merged with new chapters, reflecting the changes in practice in recent years [2,3,4,5,6].

The content is enriched through extensive illustrations, graphs, and schematics that elucidate complex concepts. This visual accompaniment renders the information accessible and comprehensible, all presented in the editor’s distinctive style, to which we have grown accustomed. Each subchapter begins with an introduction to the topic and ends with a memorable summary of key points. The book is designed to facilitate a comprehensive understanding of the field of contact lenses, beginning from the basics of visual optics and ocular anatomy and surface and progressing to conventional contact lenses and, finally, to complex and specialty lenses, dealing succinctly and clearly with the fitting, possible complications, and problem-solving, encompassing all the related issues currently known. The book notably travels beyond the mundane and contemporary, drawing attention to niche contact lens applications, such as sports. This also applies to clinical areas; the editor was aware of the significant rise in myopia and its consequences [7,8], and, as a result, the chapter on myopia control was updated.

Modern contact-fitting practice has to evaluate the patient using completely novel methods, primarily involving imagery. Understanding the currently available technology and its increasing image quality, affordability, ease of use, and portability is imperative to following complex patients and allowing co-management. Modern smartphones are an example of technology with all these characteristics, providing an attractive option for retrofitting slit-lamp systems for digital imaging, requiring minimal additional equipment. This volume provides a section dedicated to smartphone digital imaging, affirming the book’s commitment to staying current with the ever-evolving landscape [9].

*Contact Lens Practice* is an essential resource for anyone involved in fitting, managing, or even researching contact lenses, as the chapters include theoretical knowledge and clinical information, including practical tips that are immediately implementable. This makes the book a valuable reference for all eye care professionals who want to improve their clinical skills and provide better patient care. 

The book is divided into six sections, each dedicated to a specific aspect of contact lens practice. Section One provides an introduction to contact lenses, including an overview of the eye’s history, anatomy and physiology, and the principles of contact lens optics. Section Two covers soft contact lenses in great depth, such that even the most experienced practitioner can learn and be humbled by the science and complexity of products that the modern professional almost takes for granted. The section elaborates on the manufacturing, design, fitting process, materials, general optics, and characteristics of all types of soft lenses, concluding with soft lens care systems and their relationship with the lens and ocular surface. Section Three discusses rigid contact lenses; its topic format is identical to the soft lens section. The fourth section gives a comprehensive overview of the latest trends and practices in contact lens wear and care, highlighting the increased use of daily disposable lenses and the decline of lenses intended to be replaced later than monthly due to the accumulation of deposits, the loss of lens wettability, and the development of microbial contamination, addressing the environmental impact of this evolution as well. The fifth section, on special lenses, populations and their fitting considerations, provides enormous clarity and value to practitioners wanting to help these populations, the need for which has risen significantly in recent years [10]. It includes tinted lenses, toric lenses, scleral lenses for irregular corneas, hybrid lenses, the fitting process for orthokeratology lenses, and lenses for babies and small children—all with step-by-step instructions and troubleshooting tips. Prosthetic lenses require a custom fit to match the size, shape, and color of the patient’s natural eye, and this chapter discusses all this as well. The section also covers contact lenses for special populations, such as athletes or myopia control, patients with advanced ocular disease or diabetes, and post-surgery contact lenses. The final section addresses environmental concerns from proper history taking to stocking the examination room, organizing patient flow, and teaching patients insertion, removal and contact lens care. It discusses the lighting, seating, and equipment considerations that can provide an optimal learning experience for new wearers, ensuring they feel at ease during their training. The importance of proper training and education for new wearers is highlighted, along with the challenges posed by the COVID-19 pandemic and the importance of communication skills, comprehensive guidance, patience, and attention to detail to ensure safe and effective use [11]. This section discusses patient examination and management, including aftercare and complication management, even addressing compliance issues.

This new edition gives special attention to scleral contact lenses, dividing the topic into general scleral lens considerations and post-keratoplasty lenses, emphasizing the unique features to consider in each patient population. Scleral lens fitting has increased significantly [12,13], thanks in part to new technology based on Fourier transform profilometry for measuring the anterior eye surface, encompassing the corneo-scleral area, such as Eye Surface Profiler (ESP, Eagle Eye, Houten, The Netherlands) [14,15], fluorescence-based structured light topographer sMap3D (Visionary Optics, Front Royal, VA, USA) [16], and the Scheimpflug imaging technology of the corneoscleral profile (CSP) module of Pentacam (Oculus, Wetzlar, Germany) [17]. The scleral shape is a potential factor causing inferior temporal decentration in spherical scleral lenses [18], which causes poor visual quality [19]. Accurately measuring beyond the limbus has improved asymmetric contact lens designs and fitting optimization, contributing to centration [18]. As these devices become increasingly accessible to clinicians, ref. [20] the importance of this section of the book is evident.

In my opinion, the book would benefit from one more chapter on contact lenses and binocular vision. A recent comprehensive review article explores the relationship between binocular vision and contact lens discomfort [21], underscoring the intricate association between binocular vision and lens discomfort. Notably, binocular vision disorders, such as convergence insufficiency and accommodative lag, are prevalent among individuals wearing contact lenses, suggesting that contact lens use potentially exacerbates symptoms [21,22,23]. This volume might reach an even larger professional readership were it to elaborate, in more detail than it does in Section One, the potential repercussions of contact lens usage on binocular vision, such as alterations in near heterophoria and adjustments in accommodative and vergence responses [21].

To conclude, the fourth edition of the book *Contact Lens Practice* is designed to provide the latest clinically relevant information on contact lenses and their interaction with the ocular surface, making this volume quite the principal guiding authority. It is a well-written, comprehensive, and practical resource tailored to eye care professionals, and consequently, it assumes an indispensable reference point for the academic, the novice, and the experienced clinical practitioner.

## Data Availability

Not applicable.
